# Unlocking the Secrets of the Regenerating Fish Heart: Comparing Regenerative Models to Shed Light on Successful Regeneration

**DOI:** 10.3390/jcdd8010004

**Published:** 2021-01-16

**Authors:** Helen G. Potts, William T. Stockdale, Mathilda T. M. Mommersteeg

**Affiliations:** Department of Physiology, Anatomy and Genetics, University of Oxford, Oxford OX1 3PT, UK; helen.potts@new.ox.ac.uk (H.G.P.); william.stockdale@dpag.ox.ac.uk (W.T.S.)

**Keywords:** heart, regeneration, scarring, zebrafish, teleost fish, *Astyanax mexicanus*, comparative approach, interorgan

## Abstract

The adult human heart cannot repair itself after injury and, instead, forms a permanent fibrotic scar that impairs cardiac function and can lead to incurable heart failure. The zebrafish, amongst other organisms, has been extensively studied for its innate capacity to repair its heart after injury. Understanding the signals that govern successful regeneration in models such as the zebrafish will lead to the development of effective therapies that can stimulate endogenous repair in humans. To date, many studies have investigated cardiac regeneration using a reverse genetics candidate gene approach. However, this approach is limited in its ability to unbiasedly identify novel genes and signalling pathways that are essential to successful regeneration. In contrast, drawing comparisons between different models of regeneration enables unbiased screens to be performed, identifying signals that have not previously been linked to regeneration. Here, we will review in detail what has been learnt from the comparative approach, highlighting the techniques used and how these studies have influenced the field. We will also discuss what further comparisons would enhance our knowledge of successful regeneration and scarring. Finally, we focus on the *Astyanax mexicanus*, an intraspecies comparative fish model that holds great promise for revealing the secrets of the regenerating heart.

## 1. Introduction

Less than 50 years ago, there were no treatments for myocardial infarction (MI); patients were simply given painkillers and bed rest and 7 out of 10 patients did not survive. Since then, many life-saving treatments have been discovered [1,2,3] and myocardial infarction mortality rates have now fallen to 30% in the UK [4]. However, although there has been lots of progress, myocardial infarction still causes a global clinical burden [5]. During myocardial infarction, a coronary artery becomes blocked and approximately 1 billion cardiomyocytes die as they are starved of oxygen and nutrients [6]. After injury, the adult human heart can barely replace any lost cardiomyocytes and, instead, a thick, collagenous scar is deposited which cannot contract, impairs heart function and can eventually lead to incurable heart failure [7]. The only way to ‘reverse’ the effects of a heart attack and restore the heart back to its full contractile capacity is to replace the lost cardiomyocytes.

Cardiac regeneration aims to functionally restore the heart after injury. A recent regenerative strategy to achieve cardiac regeneration is to trigger the heart to repair itself. This field has exploded since two seminal papers discovered that, following injury, the zebrafish (*Danio rerio*) [8] and the neonatal mouse (*Mus musculus*) [9] can both fully repair and replace any damaged cardiac tissue with healthy new myocardium. Since these discoveries, researchers have sought to understand the cellular and molecular signals governing the innate regenerative response. It is hoped that understanding these signals will identify pathways that could be therapeutically targeted to stimulate the adult human heart to repair itself. To date, cardiac regeneration has been observed in response to a variety of insults in a range of fish, amphibian, and mammalian models [10,11].

Despite the numerous models of cardiac regeneration that have been discovered, we are no closer to stimulating regeneration in the human heart. This is largely because many studies have relied on candidate approaches and reverse genetics. In these approaches, genes, molecules or signalling pathways that are already predicted to be important in regeneration are investigated. This approach enables highly relevant genes and signalling pathways to be prioritised and tested first and, so far, candidate approach studies have successfully shown several key pathways as essential in regulating regeneration (as excellently reviewed elsewhere [12,13,14,15,16]). However, the candidate approach has a significant drawback: it is inherently biased and cannot be used to identify previously unknown pathways that may be key to unlocking the secrets of the regenerating heart.

## 2. How to Solve the Mystery of Regeneration: The Comparative Approach?

**Figure 1:**
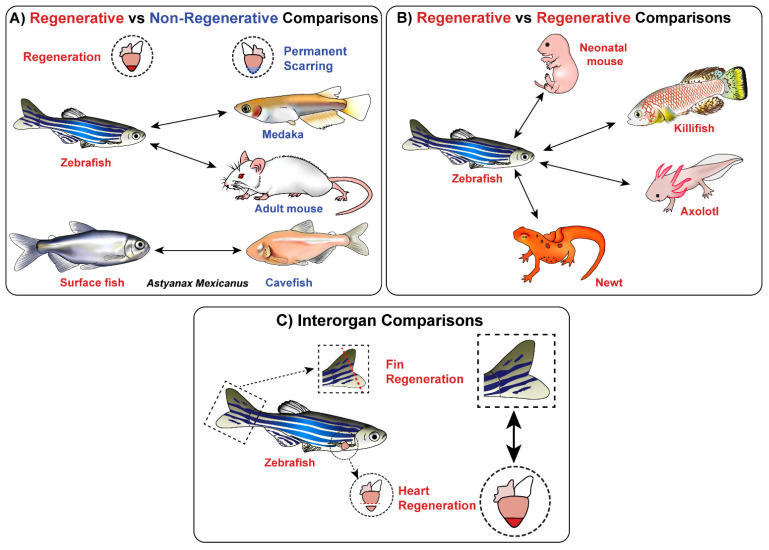
A summary of the three comparative approaches and how they have been used so far in the study of cardiac regeneration. (**A**) Interspecies comparisons between the zebrafish and the non-regenerative medaka and adult mouse have been made whilst intraspecies comparisons have been made between the regenerative and non-regenerative morphotypes of *A. mexicanus*. (**B**) Interspecies comparisons have been made between the zebrafish and the regenerative killifish, neonatal mouse, axolotl and newt. (**C**) Interorgan comparisons have been made between heart and fin regeneration in the zebrafish.

Surprisingly, very few studies have investigated cardiac regeneration using a comparative or forward genetics approach. The comparative approach directly compares at least two different models of regeneration (Figure 1). These comparisons could be between:

(1) A regenerative model versus a non-regenerative model (inter/intraspecies) to delineate the cellular and molecular differences that are driving regeneration versus permanent fibrotic scarring.

(2) A regenerative model versus a regenerative model (interspecies) to identify key pathways that have been evolutionarily conserved across species, and thus, are likely critical to successful regeneration.

(3) A regenerative heart versus a different regenerative organ (interorgan) to identify overarching regenerative programs that are driving successful, scar-free wound healing.

Utilising these three approaches holds great promise for unravelling the mysteries of the regenerating heart. Firstly, the comparative approach can uniquely identify master regulators of regeneration that are shared amongst regenerative species or amongst regenerative organs. Identifying these regenerative checkpoints could be key in developing effective therapies that can ‘switch on’ regeneration in the adult human heart. Secondly, the comparative approach is less limited by previous knowledge and can be used to identify novel pathways that have not previously been linked to regeneration. The discovery of novel regenerative signals is a rate-limiting step in reverse genetics, relying instead on serendipitous observations. Furthermore, prior knowledge has the potential to bias how we think about certain cell types or signals and their role in regeneration. In contrast, the comparative approach offers an unbiased way to screen for key genes, signalling pathways and regenerative cells. Finally, understanding the differences between successful regeneration and scarring will reveal where regeneration can fail, highlighting the key signals that need to be replicated to induce regeneration in the non-regenerative setting, and will ultimately lead to the development of an effective therapy for stimulating the adult human heart to repair itself.

In this review, we aim to explore the power of the comparative approach by focusing on what we have learnt from comparing the zebrafish to other models of regeneration. We will highlight the techniques used and what important comparisons could still be made that would bring us closer to triggering cardiac regeneration therapeutically in the adult human heart.

## 3. The Regenerative Zebrafish vs. Non-Regenerative Models of Cardiac Regeneration: How Interspecies Differences Can Pinpoint What Is Key for Regenerative Success

The best approach to be able to understand the mechanisms of successful regeneration after cardiac injury is to directly compare to a non-regenerative response, ideally in a not too distantly related species. Identifying points at which the responses to injury diverge provides an unbiased way to discover which parts of the injury response may be critically regulating regeneration. The regenerative zebrafish has so far been compared to a number of different species, from different fish models to mammalian models, which we will discuss in detail.

### 3.1. What We Can Learn from a Closely Related Teleost Fish: Medaka (Oryzias Latipes)—One of the Few Documented Non-Regenerative Fish

The zebrafish is phylogenetically closely related to the medaka, having diverged approximately 150 million years ago [17]. In contrast to the zebrafish, medaka have been found to lack the ability to regenerate their hearts; following resection injury, medaka cardiomyocytes are unable to proliferate and, instead, a persistent fibrotic scar is formed [18].

To start to understand the differential regenerative capacity of zebrafish and medaka, Lai and colleagues took a comparative approach. They directly compared bulk RNAseq data from the medaka and zebrafish post-resection injury [19]. This revealed that the immune response was a major difference between the two fish species, with the medaka showing a blunted immune response compared to the zebrafish. Quantification of the number of neutrophils and macrophages post-injury confirmed this finding and showed that macrophage wound infiltration was delayed, and fewer macrophages responded in medaka, whilst neutrophils showed a prolonged persistence at the wound. Injection of the toll-like receptor agonist poly I:C into medaka significantly accelerated macrophage recruitment and neutrophil clearance. Remarkably, they found that manipulating the medaka immune response to mirror the zebrafish led to increased regeneration in the medaka with an increase in cardiomyocyte proliferation, scar resolution and neovascularisation.

This study is a prime example of how the comparative approach can be used to unbiasedly identify a key step in the regulation of successful regeneration that, when investigated further, pinpointed macrophage and neutrophil flux as critical regulators. Their finding that replicating the regenerative immune response in a scarring model improved regeneration post-injury suggests that targeting macrophage and neutrophil dynamics could be a novel therapeutic strategy. Since this study, many groups have investigated the tight regulation of spatiotemporal leukocyte dynamics post-MI [20,21] and have shown that dysregulation inhibits regeneration and leads to fibrotic scar deposition [22,23,24]. Further characterisation of the differences in the immune response between regenerative and non-regenerative species and understanding how leukocytes regulate regeneration will be critical in developing an effective immunomodulatory therapy. In addition to highlighting leukocyte dynamics, it is very likely that additional comparisons between the zebrafish and medaka will lead to the discovery of new key players in regeneration.

### 3.2. What about the Mammalian Response to Injury: What Can We Learn from Comparing the Zebrafish to the Non-Regenerative Adult Mouse?

In contrast to the medaka, the zebrafish is only distantly related to the non-regenerative adult mouse (diverged approximately 338 million years ago [25]). There is limited work arising from this comparative model due to the large evolutionary distance and, instead, the adult mouse is typically compared to the neonate. However, transferring regenerative signals from the zebrafish into the adult mouse can be used to confirm the importance of a particular signal for successful regeneration. Honkoop et al. used this approach to confirm their finding from zebrafish that Nrg1/ErbB2 activation metabolically reprograms cardiomyocytes to shift from oxidative phosphorylation to glycolysis [26]. They showed that ErbB2 overexpression in the adult mouse was sufficient to induce cardiomyocyte proliferation in the scarring setting and improve functional recovery post-ischaemic injury. Additionally, comparing zebrafish and adult mouse microRNA dynamics post-resection and ischaemic injury has identified novel microRNAs that are downregulated in the regenerative setting but remain unchanged during scarring. Crippa et al. identified mi-R26a as a negative regulator of cardiomyocyte proliferation [27]. They showed that mi-R26a knockdown could increase the neonatal cardiomyocyte proliferative window. Similarly, Aguirre et al. discovered that adult mice fail to downregulate mi-R99/100 and let7a/c following MI, but that targeted knockdown increased functional recovery, with a significant decrease in fibrotic scarring and infarct size [28]. Cumulatively, these studies show that comparing the zebrafish and adult mouse can identify signals that increase the mammalian regenerative capacity.

In a novel comparative approach, Chen and colleagues carried out an interspecies transplantation study and injected decellularised zebrafish extracellular matrix (ECM) into the adult mouse [29]. Strikingly, they found that administration of zebrafish ECM (zECM) resulted in adult mouse cardiomyocytes re-entering the cell cycle and proliferating, leading to cardiac functional recovery and regeneration post-ischaemic injury. Characterisation of the zECM and the adult mouse ECM (mECM) showed major differences in its structural composition: mECM is highly composed of collagens, whereas the zECM contains more elastin. Their findings were remarkable, as they showed, for the first time, that transplanting regenerative tissue from a fish into a mammal could produce therapeutic benefits. In addition to elastin, recent studies have shown that the zECM comprises fibronectin [30], collagen XII [31], tenascin C [32], fibrinogens [33], periostin b [33] and fibrillin 2b [33]. Comparing the structural composition of the ECM across regenerative models, characterising how it changes with time post-injury and determining how it differs from non-regenerative models could lead to great insights in the development of a “pro-regenerative” ECM that could be used therapeutically to induce human cardiomyocytes to proliferate post-MI.

Overall, we can conclude that despite the evolutionary distance between the zebrafish and adult mouse, comparisons between these models are very useful for confirming the importance of key signals in regeneration and identifying potential therapeutic avenues for increasing the mammalian regenerative capacity.

### 3.3. What We Can Learn from Trends and Comparisons Across Multiple Species

The comparative approaches that have been discussed so far have focused on drawing comparisons between two models. While very useful, these approaches are complicated by physiological differences between the two species, as well as species unique traits. This problem can be circumvented by taking a broader approach and comparing regenerative potential across the animal kingdom.

Previous studies have suggested that heart regeneration potential and myocardial proliferation capacity are positively correlate with cardiomyocyte diploid abundance [34], whilst inducing polyploidisation in the zebrafish can inhibit regeneration [35]. Based on this, Hirose et al. utilised cardiomyocyte ploidy as an indicator of regenerative potential and, in an impressive feat, measured cardiomyocyte diploid abundance across 41 species [36]. While, understandably, they were not able to directly assess the ability for heart regeneration in all these species, which include rare animals such as the platypus, they found that cardiomyocyte diploid abundance is inversely correlated with the standard metabolic rate, body temperature and thyroid hormone levels. Thyroid hormones are major regulators of metabolism and are predicted to influence the ectotherm-to-endotherm (cold- to warm-blooded) transition during evolution [37,38]. As such, the authors proposed that polyploidisation of cardiomyocytes and the associated loss of regeneration potential is an evolutionary trade-off for the acquisition of endothermy via thyroid hormone signalling. Endothermy acquisition and an increased metabolic rate enables organisms to regulate body temperature and increase their energy production, respectively. This likely offered survival advantages in environments previously unexplored by fish and reptiles, by separating physiological performance from environment variation such as temperature [38].

To investigate whether thyroid signalling directly regulates cardiomyocyte polyploidy, Hirose and colleagues generated cardiomyocyte-specific dominant negative thyR_α_ (thyroid hormone receptor α) mice [36]. They found that loss of thyroid signalling reduced cardiomyocyte polyploidisation, which, post-ischaemia-reperfusion injury, led to increased cardiomyocyte proliferation and regeneration. In contrast, exogenous thyroid hormone exposure reduced cardiomyocyte proliferation and inhibited heart regeneration in zebrafish. Taken together, these functional experiments suggest that thyroid signalling plays a key role in cardiomyocyte ploidy, proliferation and regenerative potential. Moreover, the results provide an explanation as to why mammals have lost the ability for heart regeneration, although additional factors are likely at play.

While the approach used here is extremely compelling, there are species that are exceptions to the pattern, such as the previously described medaka, which is an ectotherm fish with non-proliferative cardiomyocytes [18]. Similarly, there is the non-regenerating Pachόn cavefish, which has a lower metabolic rate as an adaption to nutrient scarcity [39,40]. This highlights that other factors play a role in cardiomyocyte proliferation and regeneration potential, which may not be immediately observable in a multiple-species comparative approach such as this, which relies on strong trends or correlations to establish a factor underlying heart regeneration.

Nonetheless, by comparing differences in multiple species from across the animal kingdom, Hirose et al. have provided new insights into hormonal mechanisms underpinning heart regeneration. It will be fascinating to see what other potential features correlate with cardiomyocyte ploidy across multiple species. Features such as habitat oxygen levels [41,42,43,44] and the cardiac ECM composition [29] (discussed above) differ between the regenerative zebrafish and non-regenerative adult mouse: could there be a correlation with cardiomyocyte ploidy? Overall, a multi-species approach can identify key signals regulating regeneration that have been conserved across the animal kingdom with great statistical power.

## 4. Regeneration vs. Regeneration: What Evolution Can Tell Us

Many biological processes, such as metabolism [45], thermic regulation [36] and development [46], are conserved across the animal kingdom. It seems likely that the signals and genetic programs regulating cardiac regeneration will also be conserved across regenerative species. Directly searching for similarities in the injury response between different models of cardiac regeneration offers the opportunity to discover shared mechanisms that must be key in driving successful, scar-free repair.

### 4.1. What We Can Learn from the Most Commonly Used Models of Cardiac Regeneration: The Zebrafish versus the Neonatal Mouse

Despite the prevalence of the zebrafish and neonatal mouse as established models of cardiac regeneration, no studies have been carried out to date that directly compare their regenerative responses. Instead, many studies simply either confirm that environmental features, such as the oxygenation state [41], are common to both models or use a candidate approach to confirm findings from the zebrafish in the neonatal mouse (and vice versa) [47]. For example, Mahmoud et al. used such an approach to show that cardiac innervation was key to successful regeneration. Using the zebrafish, they showed that disrupting cholinergic nerve-derived signals, either by genetically reducing innervation levels or pharmacologically antagonising cholinergic receptors, led to a reduction in the number of proliferating cardiomyocytes post-resection injury in zebrafish. In order to specifically impair cholinergic nerves, they transferred their findings into the anatomically larger neonatal mouse and used mechanical cholinergic denervation. This confirmed that inhibition of cholinergic signalling impairs successful regeneration due to a decrease in cardiomyocyte proliferation [48].

The developmental, genetic, inflammatory and architectural differences between the neonatal mouse and zebrafish have probably dissuaded researchers from pursuing this comparative approach. Developmentally, the zebrafish model uses adult fish, whereas the neonatal mouse model uses pups within the first week of their life (P1–P6), whose heart is still in the growing phase. The anatomy of the regenerating zebrafish and neonatal hearts is also very different: the zebrafish has a two-chambered heart, whereas the neonatal mouse has a four-chambered heart with a much higher heart rate and blood pressure [49]. Finally, only 61.5% of mouse protein-coding genes have a corresponding zebrafish orthologue [50], which can impede comparative genomics.

Regardless of these potential barriers to the comparative approach, Simões et al. performed a complementary, side-by-side analysis of bulk RNAseq datasets from the zebrafish and neonatal mouse [51]. During their analysis, they discovered that collagens and their associated proteins were upregulated in macrophages in both the regenerative (zebrafish resection and P1 LAD ligation) and scarring (zebrafish cryoinjury and P7 LAD ligation) response to injury. To confirm macrophage collagen expression, they used in situ hybridisation to show the overlapping expression of *mpeg1* (a zebrafish macrophage marker) with the collagen *col4a1* and its associated binding protein *col4a13bpa*; both collagens were identified using the comparative analysis. Some very elegant adoptive transfer experiments showed that macrophages not only express collagens, but actively secrete them and directly contribute to scar formation in both the zebrafish and P7 mouse. Finally, they confirmed that macrophages deposit a substantial proportion of the scar as depleting macrophages of *col4a1* and *col4a3bpa* significantly decreased scar size post-injury.

Prior to this study, scars were thought to be exclusively produced by activated myofibroblasts [52]. This novel finding is particularly impressive when one considers that macrophages have long been regarded as master coordinators of tissue repair and, as such, have been subject to intense investigation [53]. Many studies have shown that macrophages are cleared by lymphatic vessels [54,55,56] and regulate CM proliferation [57], neovascularisation [58], neutrophil clearance [19] and cardiac conduction [59]. However, their direct role in scar deposition was completely unknown. This study raises the fascinating question of whether macrophages are playing any other underappreciated roles and whether collagen secretion by macrophages triggers different downstream pathways in regenerative and non-regenerative settings. Additionally, this study raises the possibility that unexpected cell types could be directly contributing to scar formation; a possibility that has been further emphasised by the finding from Koth et al. that thrombocytes also activate a myofibroblast-like gene program [60].

We are only beginning to understand the evolutionary genetic and epigenetic changes that are activated upon injury to coordinate successful regeneration. Based on our previous knowledge of how regeneration occurs in these two widely studied models, it would seem that future studies utilising the zebrafish and neonatal mouse could further identify shared interspecies regenerative programs.

### 4.2. What Can We Learn from Another Regenerative Fish: The African Killifish (Nothobranchius Furzeri)

Although multiple regenerative fish species have been discovered, no studies have sought to determine if their regenerative responses were evolutionarily conserved until recently. This year, the African killifish has been discovered as a novel vertebrate model that can regenerate its caudal fin and heart [61]. The African killifish diverged from the zebrafish approximately 230 million years ago [61]. Since divergence, both fish have faced very different strong environmental selection pressures, with the zebrafish thriving in flowing freshwater, whereas the killifish inhabit ponds that undergo annual desiccation. Wang and colleagues hypothesised that these differential environmental selection pressures and evolutionary distance would enable any shared genetic mechanisms driving regeneration to be distinguishable from species-specific mechanisms.

**Figure 2:**
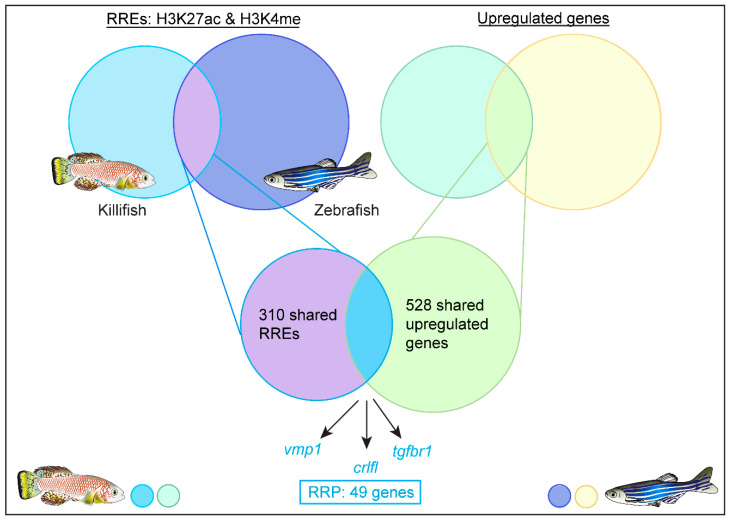
Identification of the 49 gene Regeneration Response Program (RRP).

To search for evolutionarily conserved genetic regulatory programs, they sequenced regions of DNA enriched by H3K27ac-enhancers and H3K4me-promoters using ChIPseq at baseline and 1 day after caudal fin resection. H3K27ac and H3K4me are two histone modifications that have previously been shown to mark regions of active gene expression [62,63]. Direct comparison of the ChIPseq data produced a list of putative enhancers that were activated upon injury in both fish that they coined regeneration-responsive enhancers (RREs). To determine which genes might be expressed following the activation of these RREs, they carried out RNAseq of the regenerating caudal fin. This identified 528 genes that were upregulated following injury in both the zebrafish and killifish. Comparing the shared upregulated genes with the shared RREs identified a regeneration response program (RRP) of 49 conserved genes (Figure 2), whilst single-cell RNAseq analysis showed that the RRP was upregulated specifically in regenerative blastema cells. Wang and colleagues postulated that following injury, regeneration-competent models activate the RRP by modifying their chromatin to activate the enhancers and promoters of RRP genes, resulting in upregulated expression of the RRP. Although the RRP included some genes that are already known to regulate zebrafish regeneration, such as *fgf20*, excitingly, the RRP also identified genes that had never before been linked to regeneration, such as *vmp1*, *crlfl* and *tgfbr1*. Further studies should aim to delineate what roles *vmp1, crlfl* and *tgfbr1* are playing in regulating successful regeneration.

Direct comparison of ChIPseq data from the zebrafish and killifish identified 310 RREs that were activated upon injury. Direct comparison of bulk RNAseq data after fin injury identified 528 genes that were upregulated in both the zebrafish and killifish. A final direct comparison of the 310 shared RREs and the 528 shared upregulated genes led to the identification of the RRP: a genetic program of 49 genes that is activated in response to injury to drive successful regeneration.

In addition to identifying the RRP genes themselves, Wang et al. went on to show the evolutionary importance of the RRP and its RREs. Using regenerative *(Acomys cahirinus)* and scarring *(M. musculus)* responses to ear pinna and skin injury in mouse, they showed that 20/49 RRP genes were dysregulated in regenerative-incompetent settings. They postulated that the RRP must have been subjected to evolutionary changes over time, leading to its dysregulation in animals with limited regenerative capacity. Furthermore, they showed that disruption of an RRE upstream of the killifish *inhba(2of2)* enhancer (termed K-IEN) by CRISPR/Cas9 could inhibit caudal fin and cardiac regeneration, confirming the importance of the identified RREs for successful wound healing.

To further test the evolutionary importance of the RREs, Wang et al. used mVISTA to identify K-IEN orthologues in the zebrafish (Z-IEN) and the human (H-IEN). Interestingly, they found that the pro-regenerative role of the IEN was conserved amongst the Z-IEN and the K-IEN: following injury, Z-IEN upregulated gene expression in heart and fin injury-responsive cells, and remarkably, could restore regeneration to regeneration-incompetent K-IEN^−/−^ killifish mutants. In contrast, H-IEN barely drove expression after injury and could not rescue K-IEN^−/−^ fish. This finding suggests that RREs have an ancestral evolutionary origin in teleost fish and play indispensable roles in coordinating successful wound healing.

Finally, Wang and colleagues used motif enrichment analysis to predict that the transcription factor AP-1 binds to every RRE that they identified. Inhibition of RRE:AP-1 binding, either by site-directed mutagenesis of the IEN or pharmacologically, confirmed that AP-1 binding is essential for RRE activation and successful regeneration. AP-1 has recently been shown by Beisaw and colleagues to be critical in cardiac regeneration: it regulates chromatin accessibility post-injury in the zebrafish to promote cardiomyocyte dedifferentiation, protrusion and proliferation [64]. These two studies suggest that AP-1 plays a key role in driving successful regeneration by activating pro-regenerative gene expression programs post-cryoinjury. It will be fascinating for future studies to investigate whether recapitulating AP-1:RRE binding in scarring models can “switch on” regeneration.

## 5. Can Other Regenerating Organs Give Us Insight into Cardiac Regeneration?

The comparative regeneration-competent interspecies approach assumes that signalling pathways that are critical to successful cardiac regeneration are common to all species. An intriguing question is whether regenerative programs are organ-specific or are conserved amongst different regenerative organs within the same species. The zebrafish is well suited to testing this hypothesis, as it is a very regenerative organism [65]; in addition to its heart, it can also regenerate its fins [66], hair cells [67], kidney [68], spinal cord [69], retina [70] and telencephalon [71].

Similarly to the identification of RREs regulating fin and heart regeneration [61], Pfefferli and Jawinska showed that upregulation of the *careg* element was shared amongst regeneration of the caudal fin and the heart [72]. The *careg* element is a 3.1 kB region of DNA that contains an upstream sequence of the *ctfga* gene. They found that blocking activation of the *careg* element post-injury, by inhibiting TGFβ/Activinβ signalling, inhibited regeneration; cardiomyocytes could not dedifferentiate and proliferate whilst the fin blastema failed to form. Additionally, Kang et al. identified LEN, a small intergenic enhancer element upstream of the *lepb* gene, as another enhancer that is activated during fin and heart regeneration [73]. Intriguingly, they showed that LEN-containing constructs could be designed to either promote or impair regeneration; LEN was activated upon injury to drive the expression of either pro- or anti-regenerative factors. Further work has shown that LEN also contains a 22 bp repressive element that is conserved amongst Danio species (*D. aesculapii* and *D. kyathit*) [74] and acts to spatiotemporally restrict the regenerative response to the wounded area and prevent aberrant induction [74]. This novel finding suggests that regenerative enhancers are regulated by both repressive and active elements, and the authors postulate that regeneration could be therapeutically stimulated by attenuating or activating elements of regenerative enhancers. Overall, these studies suggest that there are at least some regenerative mechanisms that are shared between fin and heart regeneration.

Unbiased, forward genetic screens could also be used to reveal genes that are shared between regenerative organs. Pei and colleagues developed a high-throughput mutagenic screening platform that identified seven genes (*hspe1, hspa13, rnpc3, smn1, gemin5, hspd1, mgat5*) that are essential for successful regeneration in zebrafish hair cells [75]. They found that these genes were also important to successful regeneration in the liver and the caudal fin. This would be an intriguing approach to apply to heart regeneration because it is not feasible to perform forward mutagenic screens of cardiac regeneration. The main barrier to forward genetics is that assessing regenerative capacity requires culling of the unique mutant so that its heart can be dissected and stained. Therefore, to prevent any mutations of interest from being lost, all mutants would need to be bred prior to screening, which would simply require the use of too many fish. To overcome this major hurdle in regenerative screens, it would be interesting to determine whether genes critical to cardiac regeneration could be learnt from mutagenesis of other zebrafish regenerative organs or from screens of impaired cardiac development [76,77]. Alternatively, the larval zebrafish heart can be injured using laser/needle-stick injuries [78]. The development of a scalable, high-throughput embryonic injury screening platform could lead to significant breakthroughs in unbiasedly identifying key genes critical for successful regeneration.

## 6. Comparative Transcriptomics: A Powerful Genetic Tool for Comparing Any Model of Regeneration

Comparative transcriptomics is a fundamental tool for studying regeneration that can easily be applied to any model organism from fish to mammals to amphibians. As we have discussed, it can identify differences in the regenerative and scarring response in the zebrafish, medaka and adult mouse. Furthermore, it can be used to highlight shared regenerative mechanisms amongst regenerative species and organs. Mercer et al. used comparative transcriptomics of DNA microarrays to show that both the zebrafish and the newt upregulate distinct ECM components and ECM-modifying proteases post-resection injury [79]. Similarly, Natarajan et al. compared bulk RNAseq after resection injury in three regenerative species: the zebrafish, the neonatal mouse and the axolotl *(Ambystoma mexicanum)* [80]. They discovered that the complement receptor C5aR1 is upregulated in all three models, whilst inhibition of C5aR1 signalling significantly impaired cardiomyocyte proliferation and impaired regeneration.

**Figure 3:**
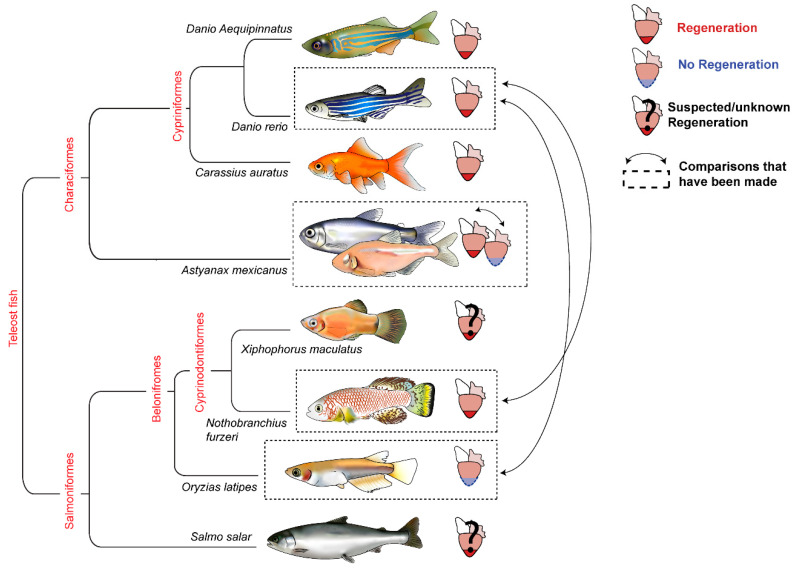
Phylogenetic tree of teleost fish models of regeneration.

Direct comparisons between closely related models are very exciting as it is easier to identify orthologous genes, enabling the application of advanced genetic techniques such as single-cell RNA sequencing. In particular, there is a lot of scope for comparative transcriptomics amongst vertebrate fish models of regeneration as many regenerative and non-regenerative models have been identified, but few comparative studies have been performed (Figure 3). It would be fascinating to determine whether reciprocal analysis between the non-regenerating cavefish of the *Astyanax mexicanus* and the zebrafish also converged on the immune system as a major difference or whether this would lead to the discovery of new critical regulators of regeneration. Furthermore, no studies have sought to determine whether the mechanisms that trigger the scarring response (instead of successful regeneration) are shared amongst non-regenerative fish species or whether there are multiple ways in which regeneration can fail.

The giant zebrafish (*D. aequipinnatus*) [81], zebrafish (*D. rerio*) [8], goldfish (*C. auratus*) [82], killifish (*N. furzeri*) [62] and the eyed surface-morph of the *A. mexicanus* [83] have all been documented to regenerate their hearts after a variety of insults such as cauterisation [84], ventricular amputation, genetic ablation [85] and cryoinjury [86]. However, despite the close relation of the zebrafish to the giant zebrafish and the goldfish, only the killifish and the zebrafish have so far been compared for shared mechanisms of regeneration [62]. The medaka (*O. latipes*) [18] and the eyeless cave-dwelling morph of *Astyanax mexicanus* [83] are the only teleost fish that form a permanent fibrotic scar following cardiac injury (although a scarring response has also been observed in the grass carp: biorXiv https://doi.org/10.1101/627752); however, to-date no comparisons of the scarring response have been made. In addition, to the above models, work on farmed salmon (*S. salar*) [87] and the Senegal birchir *(P. senegalus)* [88] suggest potential regenerative capacity whilst the the Jawinska lab are currently developing the platyfish (*X. maculat*us) as a novel model of regeneration to determine whether the scarring response seen in medaka is conserved beyond the Beloniformes order [89].

## 7. The *Astyanax mexicanus*: A Promising New Comparative Model

The *Astyanax mexicanus* was recently introduced as a new comparative model for heart regeneration, which enables the unique possibility of comparing natural fish-like regeneration with human-like scarring within the adult heart of the same species [83]. This unique feature of the *Astyanax* is made possible by the presence of two morphotypes that have not yet undergone speciation, a typical river surface fish and an eyeless cavefish. These morphotypes arose 10,000—1 million years ago when surface fish, living in rivers in northern Mexico, became separated into caves during flooding events and subsequently evolved into many different cavefish populations [90,91,92,93]. In response to cave life, they lost redundant features, such as their eyes, but also gained certain attributes beneficial for cave life [90,91,92,93,94]. Additionally, the regenerative potential of the cavefish heart changed. Stockdale et al. recently showed that, following resection injury, surface fish could regenerate their heart, while cavefish from the Pachón cave could not and formed a permanent scar, much like the adult human heart [83].

### 7.1. The Astyanax mexicanus: Comparative Transcriptomics for a Promising Comparative Model

Similar to the other comparative models described, the *Astyanax* lends itself to powerful comparative transcriptomics [83,95,96], but, uniquely, is not limited by the compromising interspecies differences seen in other models. This was showcased by Stockdale et al., where an unbiased bulk RNA-seq was used over multiple time points after injury to explore the contrasting cardiac regenerative capacities [83]. Large differences relating to the immune response, metabolism and the extracellular matrix were observed, identifying a number of genes with no known previous role in regeneration. This included *lrrc10*, which had no known role in regeneration but was significantly upregulated in the regenerating surface fish versus the cavefish after injury. In evidence of the model to detect novel genes and in a display of interspecies conservation, *lrrc10* knockout in zebrafish delayed regeneration.

### 7.2. The Astyanax mexicanus: Linking Heart Regeneration Directly to the Genome with QTL Analysis

**Figure 4:**
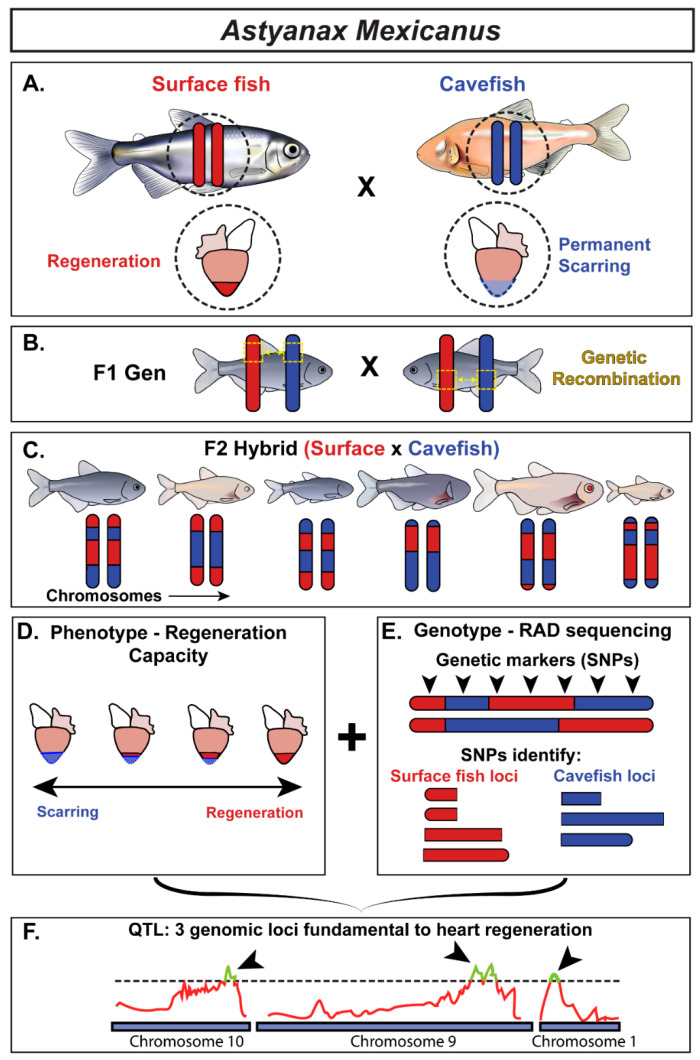
Linking heart regeneration to the genome with a quantitative trait loci (QTL) analysis in the Astyanax. (**A**) After ventricular resection, the surface fish regenerate their heart, while cavefish cannot and form a permanent scar. (**B**) Surface fish and cavefish can be interbred to form first generation offspring (F1). The F1 generation are phenotypically similar to one another and contain equal number of chromosomes from surface fish (red) and cavefish (blue). Within the F1 generation, genetic recombination (yellow boxes) causes genetic regions to be exchanged between chromosome pairs. (**C**) Crossing two F1 fish results in a second generation (F2) that is phenotypically diverse. This is caused by the genetic recombination seen in F1 fish, which leads to chromosomes of the F2 fish being a mixture of cavefish (blue) and surface fish (red) chromosomal regions. (**D**) The F2 fish also display a diverse range of heart regeneration capacity. (**E**) Regions that come from the surface fish or cavefish can be determined by restriction site-associated DNA (RAD) sequencing, which enables the detection of single nucleotide polymorphisms (SNPs) that act as genetic markers of surface or cavefish regions. (**F**) De novo linkage analysis enables heart regeneration capacity to be linked with the identified cavefish/surface fish inherited DNA regions, identifying three loci in the genome significantly linked to the degree of heart regeneration.

One of the big advantages of the *Astyanax* model is the ability to perform classical forward genetic screening methods to identify genetic loci regulating specific phenotypic changes, such as quantitative trait loci analysis (QTL) [97,98]. This is made possible because the surface fish and cavefish variants have retained the ability to interbreed, which allows generation of a fertile first-generation offspring (F1). Significantly, crossing two F1 siblings together results in a phenotypically diverse second generation (F2) hybrid. This diverse population occurs due to the exchange of genetic material between chromosomes by genetic recombination within the F1 generation, which leads to chromosomes of the F2 being a recombinant mixture of the parental cavefish/surface fish chromosomes. The F2 hybrids show mixed cavefish/surface fish features and include fish ranging from eyeless and pigmented to eyed and albino. In addition to the mixed external features, the fish show wide ranging heart regenerative capacities. The authors utilised this range of heart regenerative capacities and performed a QTL analysis, which identified three genetic loci that are linked to heart regeneration (Figure 4). Within these loci are potentially novel candidate genes fundamental to the difference between scarring and regeneration in the *Astyanax*. To help highlight candidate genes of significance, the QTL was integrated with the bulk RNAseq, which identified genes located in these loci that are differentially expressed between fish. Ultimately, all differentially expressed genes, as well as SNPs in protein coding regions or regulatory regions, are possibilities for further functional analysis.

### 7.3. The Astyanax mexicanus: Cross Species Conservation with the Zebrafish

The ability to functionally analyse genes and mechanisms that have been highlighted by the above tools is important for successful validation of a role during regeneration. The zebrafish has been the ideal model for this due to its genetic tractability and this is no different with the *Astyanax*, in which transgenesis and genome editing are also possible [99,100,101]. An additional benefit to the *Astyanax* is that it is a teleost fish, and as such, is closely related to the zebrafish [17]. Notably, the *Astyanax* is significantly more closely related to zebrafish than the comparative medaka. This evolutionary closeness means that zebrafish-specific constructs/tools can be used in the *Astyanax* [99]. As a disadvantage, the *Astyanax* take longer to reach sexual maturity than the zebrafish, resulting in an increased time to generate stable mutants/transgenics. However, this disadvantage is somewhat mitigated by the similarity with zebrafish, which makes for a high level of interspecies conservation, and the potential to investigate mechanisms and candidate genes from the *Astyanax* within the zebrafish. The strategy to investigate candidate genes in zebrafish has been employed by Stockdale et al., who used it to highlight a role in regeneration for *lrrc10*, and in a recent study by Riddle et al., who recreate a cavefish insulin receptor SNP within the zebrafish [102].

Ultimately, the value of the *Astyanax mexicanus* as a research model lies in its ability to compare differing traits within the same species, mitigating against the disadvantages present in other models. This unique aspect of the *Astyanax mexicanus* has led it to be proposed not just as a model for regeneration but also as a model for understanding traits associated with retinal degeneration and insulin resistance [97,102,103]. In terms of heart regeneration, *Astyanax mexicanus* has the potential to provide clues as to why adult mammals have lost the ability to regenerate their hearts during evolution and establish novel mechanisms underlying cardiac regeneration.

## 8. Limitations of the Comparative Approach

As we can see above, comparative models have the capacity to enhance our understanding of heart regeneration. However, as already alluded to in several models, they are not without their limitations. One of the major limiting factors is that comparisons can be confounded by both developmental (such as neonate vs. adult) and interspecies differences. Different physiologies that arise from different evolutionary selection pressures can make it difficult to identify which interspecies differences are driving differences in regenerative capacity, rather than just being an artefact of evolutionary distance. Furthermore, interspecies comparisons rely on the assumption that mechanisms regulating regeneration and scarring have been evolutionarily conserved, which may not be the case.

A secondary limitation of the comparative approach is the reliance on well-annotated, identified orthologous genes. It may not always be possible to identify orthologs for every gene due to large genomic differences. For example, the killifish/zebrafish comparative single-cell RNAseq in Wang et al. utilised only the known 1:1 orthologs between zebrafish and killifish, excluding genes from the final analysis [61]. This problem is likely to be made worse in a more evolutionary diverse comparison such as mouse to zebrafish, where only 61.5% of mouse protein-coding genes have a corresponding zebrafish ortholog [50]. However, these two limitations of interspecies differences are largely circumvented by the promising *Astyanax mexicanus* model.

An additional limitation in the comparative approach is that the injury model used has the potential to confound comparisons between regenerative responses. It has already been well documented in the zebrafish that cryoinjury and ventricular resection induced different regenerative responses: resection results in rapid scar-free healing [8], whereas cryoinjury induces scar formation and subsequent resorption [86,104,105]. Thus, when performing interspecies and interorgan comparisons, it is critical that the tissue insult being compared is as similar as possible: resection injuries should only be compared with other resections, whilst injuries that create apoptotic/necrotic tissue, such as cryoinjury and LAD ligation, should be compared with each other to mitigate this potentially confounding variable. Finally, comparisons between organs, such as the heart and fin, is an interesting approach, but this assumes that the mechanisms behind successful regeneration are conserved between organs. This assumption is somewhat inhibited by the fact that cardiac regeneration requires proliferation of pre-existing cardiomyocytes, not a progenitor population, whilst the fin relies on a lineage restricted proliferative progenitor population, which forms a structure called a blastema [106,107,108]. Moreover, these cell types driving regeneration in their respective tissue are very different in their structure, size and metabolism. This brings in the question: do these diverse cell types activate common mechanisms required for the regenerative response? It is likely that not all mechanisms are conserved between organs, and as such, this model can only detect mechanisms that are conserved.

## 9. Conclusions

Since the discovery that zebrafish can regenerate their hearts following amputation, huge progress has been made in understanding the key signals that regulate successful regeneration. However, to achieve the ultimate goal of cardiac regeneration and stimulate the adult human heart to repair itself, a combination of comparative and candidate studies will be required. Comparisons of regenerative species will highlight master regulators that are activated upon injury to ‘switch on’ regeneration, whilst comparisons between regenerative and non-regenerative species will reveal which signals need to be replicated in the scarring setting in order to induce regeneration. Signals identified by comparative studies should then be the focus of follow up candidate approaches to determine the exact role a key signal or gene plays in regeneration and to investigate how this signal can be manipulated in order to stimulate human heart regeneration.

## Data Availability

Data sharing not applicable.

## References

[1] Baigent C., Collins R., Appleby P., Parish S., Sleight P., Peto R. (1998). ISIS-2: 10 year survival among patients with suspected acute myocardial infarction in randomised comparison of intravenous streptokinase, oral aspirin, both, or neither. BMJ.

[2] Grüntzig A.R., Senning Å., Siegenthaler W.E. (1979). Nonoperative Dilatation of Coronary-Artery Stenosis. N. Engl. J. Med..

[3] Melly L., Torregrossa G., Lee T., Jansens J.L., Puskas J.D. (2018). Fifty years of coronary artery bypass grafting. J. Thorac. Dis..

[4] British Heart Foundation (2020). UK Factsheet. Br. Heart Found..

[5] Khan M., Hashim M.J., Mustafa H., Baniyas M.Y., Suwaidi S.K.B.M.A., AlKatheeri R., Alblooshi F.M.K., Almatrooshi M.E.A.H., Alzaabi M.E.H., Darmaki R.S.A. (2020). Global Epidemiology of Ischemic Heart Disease: Results from the Global Burden of Disease Study. Cureus.

[6] Gerbin K.A., Murry C.E. (2015). The winding road to regenerating the human heart. Cardiovascular Pathology.

[7] Prabhu S.D., Frangogiannis N.G. (2016). The Biological Basis for Cardiac Repair After Myocardial Infarction: From Inflammation to Fibrosis. Circ. Res..

[8] Poss K.D., Wilson L.G., Keating M.T. (2002). Heart Regeneration in Zebrafish. Science.

[9] Porrello E.R., Mahmoud A.I., Simpson E., Hill J.A., Richardson J.A., Olson E.N., Sadek H. (2011). Transient Regenerative Potential of the Neonatal Mouse Heart. Science.

[10] Price E.L., Vieira J.M., Riley P.R. (2019). Model organisms at the heart of regeneration. DMM Dis. Models Mech..

[11] Vivien C.J., Hudson J.E., Porrello E.R. (2016). Evolution, comparative biology and ontogeny of vertebrate heart regeneration. NPJ Regen. Med..

[12] Cahill T.J., Choudhury R.P., Riley P.R. (2017). Heart regeneration and repair after myocardial infarction: Translational opportunities for novel therapeutics. Nat. Rev. Drug Discov..

[13] Uygur A., Lee R.T. (2016). Mechanisms of Cardiac Regeneration. Dev. Cell.

[14] Broughton K.M., Wang B.J., Firouzi F., Khalafalla F., Dimmeler S., Fernandez-Aviles F., Sussman M.A. (2018). Mechanisms of Cardiac Repair and Regeneration. Circ. Res..

[15] Porrello E.R., Olson E.N. (2014). A neonatal blueprint for cardiac regeneration. Stem Cell Res..

[16] González-Rosa J.M., Burns C.E., Burns C.G., Burns C.C.G. (2017). Zebrafish heart regeneration: 15 years of discoveries. Regeneration.

[17] Kirchmaier S., Naruse K., Wittbrodt J., Loosli F. (2015). The Genomic and Genetic Toolbox of the Teleost Medaka (Oryzias latipes). Genetics.

[18] Ito K., Morioka M., Kimura S., Tasaki M., Inohaya K., Kudo A. (2014). Differential reparative phenotypes between zebrafish and medaka after cardiac injury. Dev. Dyn..

[19] Lai S.-L., Marín-Juez R., Moura P.L., Kuenne C., Lai J.K.H., Tsedeke A.T., Guenther S., Looso M., Stainier D.Y.R. (2017). Reciprocal analyses in zebrafish and medaka reveal that harnessing the immune response promotes cardiac regeneration. ELife.

[20] Bevan L., Lim Z.W., Venkatesh B., Riley P.R., Martin P., Richardson R.J. (2020). Specific macrophage populations promote both cardiac scar deposition and subsequent resolution in adult zebrafish. Cardiovasc. Res..

[21] Hui S.P., Sheng D.Z., Sugimoto K., Gonzalez-Rajal A., Nakagawa S., Hesselson D., Kikuchi K. (2017). Zebrafish Regulatory T Cells Mediate Organ-Specific Regenerative Programs. Dev. Cell.

[22] Xu S., Xie F., Tian L., Manno S.H.C., Manno F.A., Cheng S.H. (2019). Prolonged neutrophil retention in the wound impairs zebrafish heart regeneration after cryoinjury. Fish Shellfish. Immunol..

[23] Godwin J.W., Debuque R., Salimova E., Rosenthal N.A. (2017). Heart regeneration in the salamander relies on macrophage-mediated control of fibroblast activation and the extracellular landscape. NPJ Regen. Med..

[24] Sanz-Morejón A., García-Redondo A.B., Reuter H., Marques I.J., Bates T., Galardi-Castilla M., Große A., Manig S., Langa X., Ernst A. (2019). Wilms Tumor 1b Expression Defines a Pro-regenerative Macrophage Subtype and Is Required for Organ Regeneration in the Zebrafish. Cell Rep..

[25] Liu T.X., Zhou Y., Kanki J.P., Deng M., Rhodes J., Yang H.W., Sheng X.M., Zon L.I., Look A.T. (2002). Evolutionary conservation of zebrafish linkage group 14 with frequently deleted regions of human chromosome 5 in myeloid malignancies. Proc. Natl. Acad. Sci. USA.

[26] Honkoop H., de Bakker D.E., Aharonov A., Kruse F., Shakked A., Nguyen P.D., de Heus C., Garric L., Muraro M.J., Shoffner A. (2019). Single-cell analysis uncovers that metabolic reprogramming by ErbB2 signaling is essential for cardiomyocyte proliferation in the regenerating heart. Elife.

[27] Crippa S., Nemir M., Ounzain S., Ibberson M., Berthonneche C., Sarre A., Boisset G., Maison D., Harshman K., Xenarios I. (2016). Comparative transcriptome profiling of the injured zebrafish and mouse hearts identifies miRNA-dependent repair pathways. Cardiovasc. Res..

[28] Aguirre A., Montserrat N., Zacchigna S., Nivet E., Hishida T., Krause M.N., Kurian L., Ocampo A., Vazquez-Ferrer E., Rodriguez-Esteban C. (2014). In vivo activation of a conserved microRNA program induces robust mammalian heart regeneration. Cell Stem Cell.

[29] Chen W.C.W., Wang Z., Missinato M.A., Park D.W., Long D.W., Liu H.-J., Zeng X., Yates N.A., Kim K., Wang Y. (2016). Decellularized zebrafish cardiac extracellular matrix induces mammalian heart regeneration. Sci. Adv..

[30] Wang J., Karra R., Dickson A.L., Poss K.D. (2013). Fibronectin is deposited by injury-activated epicardial cells and is necessary for zebrafish heart regeneration. Dev. Biol..

[31] Marro J., Pfefferli C., Charles A.-S.D.P., Bise T., Jazwinska A. (2016). Collagen XII Contributes to Epicardial and Connective Tissues in the Zebrafish Heart during Ontogenesis and Regeneration. PLoS ONE.

[32] Chablais F., Jaźwińska A. (2012). The regenerative capacity of the zebrafish heart is dependent on TGFβ signaling. Development.

[33] Garcia-Puig A., Mosquera J.L., Jiménez-Delgado S., García-Pastor C., Jorba I., Navajas D., Canals F., Raya A. (2019). Proteomics Analysis of Extracellular Matrix Remodeling During Zebrafish Heart Regeneration. Mol. Cell. Proteom..

[34] Patterson M., Barske L., Van Handel B., Rau C.D., Gan P., Sharma A., Parikh S., Denholtz M., Huang Y., Yamaguchi Y. (2017). Frequency of mononuclear diploid cardiomyocytes underlies natural variation in heart regeneration. Nat. Genet..

[35] González-Rosa J.M., Sharpe M., Field D., Soonpaa M.H., Field L.J., Burns C.E. (2018). Myocardial Polyploidization Creates a Barrier to Heart Regeneration in Zebrafish. Dev. Cell.

[36] Hirose K., Payumo A.Y., Cutie S., Hoang A., Zhang H., Guyot R., Lunn D., Bigley R.B., Yu H., Wang J. (2019). Evidence for hormonal control of heart regenerative capacity during endothermy acquisition. Science.

[37] Hulbert A.J. (2000). Thyroid hormones and their effects: A new perspective. Biol. Rev. Camb. Philos. Soc..

[38] Little A.G., Seebacher F. (2014). The evolution of endothermy is explained by thyroid hormonemediated responses to cold in early vertebrates. J. Exp. Biol..

[39] Moran D., Softley R., Warrant E.J. (2014). Eyeless Mexican Cavefish Save Energy by Eliminating the Circadian Rhythm in Metabolism. PLoS ONE.

[40] Aspirasa A.C., Rohnera N., Martineaua B., Borowskyb R.L., Tabina C.J. (2015). Melanocortin 4 receptor mutations contribute to the adaptation of cavefish to nutrient-poor conditions. Proc. Natl. Acad. Sci. USA.

[41] Puente B.N., Kimura W., Muralidhar S.A., Moon J., Amatruda J.F., Phelps K.L., Grinsfelder D., Rothermel B.A., Chen R., Garcia J.A. (2014). The oxygen-rich postnatal environment induces cardiomyocyte cell-cycle arrest through DNA damage response. Cell.

[42] Webster W.S., Abela D. (2007). The effect of hypoxia in development. Birth Defects Res. Part C Embryo Today Rev..

[43] Rees B.B., Sudradjat F.A., Love J.W. (2001). Acclimation to hypoxia increases survival time of zebrafish, Danio rerio, during lethal hypoxia. J. Exp. Zoöl..

[44] Roesner A., Hankeln T., Burmester T. (2006). Hypoxia induces a complex response of globin expression in zebrafish (Danio rerio). J. Exp. Biol..

[45] Peregrín-Alvarez J.M., Sanford C., Parkinson J. (2009). The conservation and evolutionary modularity of metabolism. Genome Biol..

[46] Santini S., Boore J.L., Meyer A. (2003). Evolutionary Conservation of Regulatory Elements in Vertebrate Hox Gene Clusters. Genome Res..

[47] Singh B.N., Koyano-Nakagawa N., Gong W., Moskowitz I.P., Weaver C.V., Braunlin E., Das S., Van Berlo J.H., Garry M.G., Garry D.J. (2018). A conserved HH-Gli1-Mycn network regulates heart regeneration from newt to human. Nat. Commun..

[48] Mahmoud A.I., O’Meara C.C., Gemberling M., Zhao L., Bryant D.M., Zheng R., Gannon J.B., Cai L., Choi W.-Y., Egnaczyk G.F. (2015). Nerves Regulate Cardiomyocyte Proliferation and Heart Regeneration. Dev. Cell.

[49] Rubin N., Harrison M.R., Krainock M., Kim R., Lien C.-L. (2016). Recent advancements in understanding endogenous heart regeneration—insights from adult zebrafish and neonatal mice. Semin. Cell Dev. Biol..

[50] Giffen K.P., Liu H., Kramer K.L., He D.Z.Z. (2019). Expression of Protein-Coding Gene Orthologs in Zebrafish and Mouse Inner Ear Non-sensory Supporting Cells. Front. Neurosci..

[51] Simões F.C., Cahill T.J., Kenyon A., Gavriouchkina D., Vieira J.M., Sun X., Pezzolla D., Ravaud C., Masmanian E., Weinberger M. (2020). Macrophages directly contribute collagen to scar formation during zebrafish heart regeneration and mouse heart repair. Nat. Commun..

[52] Travers J.G., Kamal F.A., Robbins J., Yutzey K.E., Blaxall B.C. (2016). Cardiac fibrosis: The fibroblast awakens. Circ. Res..

[53] Krzyszczyk P., Schloss R., Palmer A., Berthiaume F. (2018). The role of macrophages in acute and chronic wound healing and interventions to promote pro-wound healing phenotypes. Front. Physiol..

[54] Harrison M.R., Feng X., Mo G., Aguayo A., Villafuerte J., Yoshida T., A Pearson C., Schulte-Merker S., Lien C.-L. (2019). Late developing cardiac lymphatic vasculature supports adult zebrafish heart function and regeneration. eLife.

[55] Gancz D., Raftrey B.C., Perlmoter G., Marín-Juez R., Semo J., Matsuoka R.L., Karra R., Raviv H., Moshe N., Addadi Y. (2019). Distinct origins and molecular mechanisms contribute to lymphatic formation during cardiac growth and regeneration. Elife.

[56] Vivien C.J., Pichol-Thievend C., Sim C.B., Smith L.M., Bower N.I., Hogan B.M., Hudson J.E., Francois M., Porrello E.R. (2019). Vegfc/d-dependent regulation of the lymphatic vasculature during cardiac regeneration is influenced by injury context. NPJ Regen. Med..

[57] LaVine K.J., Epelman S., Uchida K., Weber K.J., Nichols C.G., Schilling J.D., Ornitz D.M., Randolph G.J., Mann D.L. (2014). Distinct macrophage lineages contribute to disparate patterns of cardiac recovery and remodeling in the neonatal and adult heart. Proc. Natl. Acad. Sci. USA.

[58] Aurora A.B., Porrello E.R., Tan W., Mahmoud A.I., Hill J.A., Bassel-Duby R., Sadek H.A., Olson E.N. (2014). Macrophages are required for neonatal heart regeneration. J. Clin. Investig..

[59] Hulsmans M., Clauss S., Xiao L., Aguirre A.D., King K.R., Hanley A., Hucker W.J., Wülfers E.M., Seemann G., Courties G. (2017). Macrophages Facilitate Electrical Conduction in the Heart. Cell.

[60] Koth J., Wang X., Killen A.C., Stockdale W.T., Potts H.G., Jefferson A., Bonkhofer F., Riley P.R., Patient R.K., Göttgens B. (2020). Runx1 promotes scar deposition and inhibits myocardial proliferation and survival during zebrafish heart regeneration. Development.

[61] Wang W., Hu C.-K., Zeng A., Alegre D., Hu D., Gotting K., Granillo A.O., Wang Y., Robb S.M., Schnittker R. (2020). Changes in regeneration-responsive enhancers shape regenerative capacities in vertebrates. Science.

[62] Creyghton M.P., Cheng A.W., Welstead G.G., Kooistra T.G., Carey B.W., Steine E.J., Hanna J., Lodato M.A., Frampton G.M., Sharp P.A. (2010). Histone H3K27ac separates active from poised enhancers and predicts developmental state. Proc. Natl. Acad. Sci. USA.

[63] Shilatifard A. (2012). The COMPASS Family of Histone H3K4 Methylases: Mechanisms of Regulation in Development and Disease Pathogenesis. Annu. Rev. Biochem..

[64] Beisaw A., Kuenne C., Guenther S., Dallmann J., Wu C.-C., Bentsen M., Looso M., Stainier D.Y. (2020). AP-1 Contributes to Chromatin Accessibility to Promote Sarcomere Disassembly and Cardiomyocyte Protrusion During Zebrafish Heart Regeneration. Circ. Res..

[65] Beffagna G. (2019). Zebrafish as a Smart Model to Understand Regeneration After Heart Injury: How Fish Could Help Humans. Front. Cardiovasc. Med..

[66] Poss K.D., Keating M.T., Nechiporuk A. (2003). Tales of regeneration in zebrafish. Dev. Dyn..

[67] Thomas E.D., Cruz I.A., Hailey D.W., Raible D.W. (2015). There and back again: Development and regeneration of the zebrafish lateral line system. Wiley Interdiscip. Rev. Dev. Biol..

[68] Diep C.Q., Ma D., Deo R.C., Holm T.M., Naylor R.W., Arora N., Wingert R.A., Bollig F., Djordjevic G., Lichman B. (2011). Identification of adult nephron progenitors capable of kidney regeneration in zebrafish. Nat. Cell Biol..

[69] Becker T., Wullimann M.F., Becker C.G., Bernhardt R.R., Schachner M. (1997). Axonal regrowth after spinal cord transection in adult zebrafish. J. Comp. Neurol..

[70] Vihtelic T.S., Hyde D.R. (2000). Light-induced rod and cone cell death and regeneration in the adult albino zebrafish (Danio rerio) retina. J. Neurobiol..

[71] Kroehne V., Freudenreich D., Hans S., Kaslin J., Brand M. (2011). Regeneration of the adult zebrafish brain from neurogenic radial glia-type progenitors. Development.

[72] Pfefferli C., Jazwinska A. (2017). The careg element reveals a common regulation of regeneration in the zebrafish myocardium and fin. Nat. Commun..

[73] Kang J., Hu J., Karra R., Dickson A.L., Tornini V.A., Nachtrab G., Gemberling M., Goldman J.A., Black B.L., Poss K.D. (2016). Modulation of tissue repair by regeneration enhancer elements. Nature.

[74] Begeman I.J., Shin K., Osorio-Méndez D., Kurth A., Lee N., Chamberlain T.J., Pelegri F.J., Kang J. (2020). Decoding an organ regeneration switch by dissecting cardiac regeneration enhancers. Development.

[75] Pei W., Xu L., Huang S.C., Pettie K.P., Idol J., Rissone A., Jimenez E., Sinclair J.W., Slevin C., Varshney G.K. (2018). Guided genetic screen to identify genes essential in the regeneration of hair cells and other tissues. NPJ Regen. Med..

[76] Yu Q., Shen Y., Chatterjee B., Siegfried B.H., Leatherbury L., Rosenthal J., Lucas J.F., Wessels A., Spurney C.F., Wu Y.-J. (2004). ENU induced mutations causing congenital cardiovascular anomalies. Development.

[77] Smith K.A., Lagendijk A.K., Courtney A.D., Chen H., Paterson S., Hogan B.M., Wicking C., Bakkers J. (2011). Transmembrane protein 2 (Tmem2) is required to regionally restrict atrioventricular canal boundary and endocardial cushion development. Development.

[78] Evans M.A., Smart N., Dubé K.N., Bollini S., Clark J.E., Evans H.G., Taams L.S., Richardson R.J., Lévesque M., Martin P. (2013). Thymosin β4-sulfoxide attenuates inflammatory cell infiltration and promotes cardiac wound healing. Nat. Commun..

[79] Mercer S.E., Odelberg S.J., Simon H. (2013). A dynamic spatiotemporal extracellular matrix facilitates epicardial-mediated vertebrate heart regeneration. Dev. Biol..

[80] Natarajan N., Abbas Y., Bryant D.M., Gonzalez-Rosa J.M., Sharpe M., Uygur A., Cocco-Delgado L.H., Ho N.N., Gerard N.P., Gerard C. (2018). Complement Receptor C5aR1 Plays an Evolutionarily Conserved Role in Successful Cardiac Regeneration. Circulation.

[81] Lafontant P., Burns A.R., Grivas J.A., Lesch M.A., Lala T.D., Reuter S.P., Field L.J., Frounfelter T.D. (2012). The Giant Danio (D. Aequipinnatus) as A Model of Cardiac Remodeling and Regeneration. Anat. Rec. Adv. Integr. Anat. Evol. Biol..

[82] Grivas J., Haag M., Johnson A., Manalo T., Roell J., Das T.L., Brown E., Burns A.R., Lafontant P. (2014). Cardiac repair and regenerative potential in the goldfish (Carassius auratus) heart. Comp. Biochem. Physiol. Part C Toxicol. Pharmacol..

[83] Stockdale W.T., Lemieux M.E., Killen A.C., Zhao J., Hu Z., Riepsaame J., Hamilton N., Kudoh T., Riley P.R., Van Aerle R. (2018). Heart Regeneration in the Mexican Cavefish. Cell Rep..

[84] Van Dyck P.K., Hockaden N., Nelson E.C., Koch A.R., Hester K.L., Pillai N., Coffing G.C., Burns A.R., Lafontant P. (2020). Cauterization as a Simple Method for Regeneration Studies in the Zebrafish Heart. J. Cardiovasc. Dev. Dis..

[85] Wang J., Panáková D., Kikuchi K., Holdway J.E., Gemberling M., Burris J.S., Singh S.P., Dickson A.L., Lin Y.-F., Sabeh M.K. (2011). The regenerative capacity of zebrafish reverses cardiac failure caused by genetic cardiomyocyte depletion. Development.

[86] Chablais F., Veit J., Rainer G., Jazwinska A. (2011). The zebrafish heart regenerates after cryoinjury-induced myocardial infarction. BMC Dev. Biol..

[87] Ferguson H.W., Kongtorp R.T., Taksdal T., Graham D., Falk K. (2005). An outbreak of disease resembling heart and skeletal muscle inflammation in Scottish farmed salmon, Salmo salar L., with observations on myocardial regeneration. J. Fish Dis..

[88] Kikuchi K., Holdway J.E., Major R.J., Blum N., Dahn R.D., Begemann G., Poss K.D. (2011). Retinoic Acid Production by Endocardium and Epicardium Is an Injury Response Essential for Zebrafish Heart Regeneration. Dev. Cell.

[89] Jazwinska A., Blanchoud S. (2020). Towards deciphering variations of heart regeneration in fish. Curr. Opin. Physiol..

[90] Herman A., Brandvain Y., Weagley J., Jeffery W.R., Keene A.C., Kono T.J.Y., Bilandžija H., Borowsky R., Espinasa L., O’Quin K. (2018). The role of gene flow in rapid and repeated evolution of cave-related traits in Mexican tetra, Astyanax mexicanus. Mol. Ecol..

[91] Fumey J., Hinaux H., Noirot C., Thermes C., Rétaux S., Casane D. (2018). Evidence for late Pleistocene origin of Astyanax mexicanus cavefish. BMC Evol. Biol..

[92] Gross J.B., Meyer B., Perkins M. (2015). The rise of Astyanax cavefish. Dev. Dyn..

[93] Gross J.B. (2012). The complex origin of Astyanax cavefish. BMC Evol. Biol..

[94] Jeffery W.R. (2009). Regressive Evolution inAstyanaxCavefish. Annu. Rev. Genet..

[95] Krishnan J., Persons J.L., Peuss R., Hassan H., Kenzior A., Xiong S., Olsen L., Maldonado E., Kowalko J.E., Rohner N. (2020). Comparative transcriptome analysis of wild and lab populations of Astyanax mexicanus uncovers differential effects of environment and morphotype on gene expression. J. Exp. Zoöl. Part B Mol. Dev. Evol..

[96] Peuß R., Box A.C., Chen S., Wang Y., Tsuchiya D., Persons J.L., Kenzior A., Maldonado E., Krishnan J., Scharsack J.P. (2020). Adaptation to low parasite abundance affects immune investment and immunopathological responses of cavefish. Nat. Ecol. Evol..

[97] O’Quin K.E., Yoshizawa M., Doshi P., Jeffery W.R. (2013). Quantitative Genetic Analysis of Retinal Degeneration in the Blind Cavefish Astyanax mexicanus. PLoS ONE.

[98] Protas M.E., Hersey C., Kochanek D., Zhou Y., Wilkens H., Jeffery W.R., Zon L.I., Borowsky R., Tabin C.J. (2005). Genetic analysis of cavefish reveals molecular convergence in the evolution of albinism. Nat. Genet..

[99] Stahl B.A., Peuß R., McDole B., Kenzior A., Jaggard J.B., Gaudenz K., Krishnan J., McGaugh S.E., Duboue E.R., Keene A.C. (2019). Stable transgenesis in Astyanax mexicanus using the Tol2 transposase system. Dev. Dyn..

[100] Kowalko J.E., Ma L., Jeffery W.R. (2016). Genome Editing in Astyanax mexicanus Using Transcription Activator-like Effector Nucleases (TALENs). J. Vis. Exp..

[101] Klaassen H., Wang Y., Adamski K., Rohner N., Kowalko J.E. (2018). CRISPR mutagenesis confirms the role of oca2 in melanin pigmentation in Astyanax mexicanus. Dev. Biol..

[102] Riddle M.R., Aspiras A.C., Gaudenz K., Peuß R., Sung J.Y., Martineau B., Peavey M., Box A.C., Tabin J.A., McGaugh S. (2018). Insulin resistance in cavefish as an adaptation to a nutrient-limited environment. Yearb. Paediatr. Endocrinol..

[103] Albertson R.C., Cresko W., Detrich H.W., Postlethwait J.H. (2009). Evolutionary mutant models for human disease. Trends Genet..

[104] Schnabel K., Wu C.-C., Kurth T., Weidinger G. (2011). Regeneration of Cryoinjury Induced Necrotic Heart Lesions in Zebrafish Is Associated with Epicardial Activation and Cardiomyocyte Proliferation. PLoS ONE.

[105] González-Rosa J.M., Martín V., Peralta M., Torres M., Mercader N. (2011). Extensive scar formation and regression during heart regeneration after cryoinjury in zebrafish. Development.

[106] Kikuchi K., Holdway J.E., Werdich A.A., Anderson R.M., Fang Y., Egnaczyk G.F., Evans T., Macrae C.A., Stainier D.Y.R., Poss K.D. (2010). Primary contribution to zebrafish heart regeneration by gata4+ cardiomyocytes. Nat. Cell Biol..

[107] Jopling C., Sleep E., Raya M., Martí M., Raya A., Belmonte J.C. (2010). Zebrafish heart regeneration occurs by cardiomyocyte dedifferentiation and proliferation. Nature.

[108] Sehring I.M., Weidinger G. (2019). Recent advancements in understanding fin regeneration in zebrafish. Wiley Interdiscip. Rev. Dev. Biol..

